# Role of *Rhizophagus intraradices* in Mitigating Salt Stress of *Sulla carnosa* Through Modulating Plant Hormones (ABA, SA, and JA) and Nutrient Profile

**DOI:** 10.3390/biology14040341

**Published:** 2025-03-26

**Authors:** Rabaa Hidri, Walid Zorrig, Ahmed Debez, Ouissal Metoui-Ben Mahmoud, Angel María Zamarreño, José María García-Mina, Salma Nait Mohamed, Chedly Abdelly, Rosario Azcon, Ricardo Aroca

**Affiliations:** 1 Laboratory of Extremophile Plants, Centre of Biotechnology of Borj-Cedria, P.O. Box 901, Hammam-Lif 2050, Tunisia; hidrirabaa@gmail.com (R.H.); zorrigwalid@gmail.com (W.Z.); ahmed_debez@yahoo.com (A.D.); wissal.benmahmoud@cbbc.rnrt.tn (O.M.-B.M.); abdelly.chedly@gmail.com (C.A.); 2 Biodiversity and Environmental Institute, BIOMA, University of Navarra, Irunlarrea 1, 31008 Pamplona, Spain; angelmarizama@unav.es (A.M.Z.);; 3 Laboratory of Olive Biotechnology, Centre of Biotechnology of Borj-Cedria, P.O. Box 901, Hammam-Lif 2050, Tunisia; salma.nait@cbbc.rnrt.tn; 4 Department of Soil and Plant Microbiology, Zaidin Experimental Station (CSIC), C/Profesor Albareda 1, 18008 Granada, Spain; rosario.azcon@eez.csic.es

**Keywords:** arbuscular mycorrhizal fungi, signaling hormones, abscisic acid, salicylic acid, jasmonic acid, nutrient contents

## Abstract

Salt accumulation can degrade soil properties, decrease its productivity, and harm its ecological functions. Introducing salt-tolerant plant species associated with arbuscular mycorrhizal fungi (AMF) can act as an effective biological method for restoring salinized soils. AMF colonize plant roots and improve their nutrient acquisition capacity. However, there is limited knowledge on how AMF affects the production of signaling molecules, e.g., abscisic acid (ABA), salicylic acid (SA), and jasmonic acid (JA), related to plant–microbe interactions under salinity. Here, we assess the potential benefits of the AMF *Rhizophagus intraradices* in enhancing plant growth and nutrient uptake in addition to modulating stress hormone signaling levels (ABA, SA, and JA) of the facultative halophyte *Sulla carnosa* under saline conditions. Plants were grown in pots filled with soil and irrigated with 200 mM NaCl for 1 month. AMF symbiosis substantially increased the shoot dry weight (+107%), root dry weight (+67%), photosynthetic pigment content (chlorophyll a, chlorophyll b, and carotenoids), and nutrient uptake (C, N, P, K, Cu, and Zn) while significantly limiting the increase in the shoot Na^+^ concentration and H_2_O_2_ content caused by salinity stress. Mycorrhizal symbiosis significantly enhanced the root and shoot SA levels by 450% and 32%, respectively, compared to the stressed non-inoculated plants, potentially contributing to enhanced systemic resistance and osmotic adjustment under saline conditions. Salt stress increased the shoot ABA content, especially in *R. intraradices*-inoculated plants (113% higher than in stressed non-mycorrhizal plants). These findings confirm that AMF mitigated the adverse effects of salinity on *S. carnosa* by increasing the SA and ABA levels and reducing oxidative damage.

## Simple Summary

Soil salinity is becoming one of the most restrictive factors in crop production. To address this challenge, one solution is the use of beneficial soil microorganisms, such as arbuscular mycorrhizal fungi. Here, we describe how plants inoculated with an arbuscular mycorrhizal fungus grow better under saline conditions, thanks in part to the regulation of plant hormone levels (abscisic acid and salicylic acid) and improved nutrient uptake by roots. These results enhance our understanding of how arbuscular mycorrhizal fungi help plants tolerate salinity and highlight their potential as a valuable tool for improving crop yield under saline conditions.

## 1. Introduction

Soil salinization is one of the most harmful environmental stresses, hindering vegetation restoration by negatively affecting plant growth and development [1]. The application of salt-tolerant plant species in the vegetation restoration of saline soil has received increasing attention [2]. The introduction of some microorganisms in the rhizosphere, especially arbuscular mycorrhizal fungi (AMF), is useful in developing strategies to improve plant performance in saline soils [2]. AM fungi have a significant impact on maintaining soil health in saline areas and helping plants adapt to salt stress [3]. Therefore, the reintroduction of salt-tolerant plants, combined with the effective management of fungi, serves as a valuable biotechnological tool for restoring salt-affected soils. AMF are common in saline soil [4] and have been shown to substantially improve the salt tolerance of host plants [5,6,7]. To mitigate salt stress, AMF employ mechanisms such as improving water uptake, increasing mineral uptake, especially of phosphorus (P) [8,9], balancing ion homeostasis [10], improving antioxidant systems [6,9,10], boosting the plant capacity to produce chlorophyll and conduct photosynthesis [11], and improving osmotic adjustment in plants [7,12]. In addition, AM symbiosis can also mitigate element toxicity by restricting and/or excluding the entry of salt ions into various plants, soil, or fungal compartments, as well as by releasing glomalin-related proteins into the soil in saline environments [7,13,14]. Glomalin plays a crucial role in soil carbon storage by stabilizing soil aggregates and also represents a potentially important soil carbon pool [15].

Plant-associated microorganisms can improve plant growth under salinity stress by influencing the host plants’ hormonal balance, i.e., modulating the levels of endogenous phytohormones in the plant tissues [16]. Extensive research has been conducted on how plant growth-promoting bacteria regulate phytohormones during salt stress [17,18,19,20,21]. However, less attention has been given to how AMF affect stress hormone signaling in host plants under salinity stress [22,23,24]. Plants initiate complex signaling pathways to cope with both biotic and abiotic stresses. Among the signaling molecules, abscisic acid (ABA), jasmonic acid (JA), and salicylic acid (SA) are particularly important due to their role in regulating plant responses to abiotic stress, often through extensive signaling cross-talk with other plant growth regulators [25].

ABA is considered the major stress hormone that coordinates plant responses to challenging environments, playing a crucial role in signaling salt stress and adaptation by transducing environmental signals into changes in gene expression [26,27,28]. Studies have revealed that ABA improves the plant’s ability to manage salt stress through various mechanisms, such as reducing stomatal conductance, which prevents water loss through transpiration, altering root structure, and inducing a wide range of gene expression and physiological changes [29]. Furthermore, ABA is essential in regulating the uptake of salt ions and facilitating the transport of Na^+^ and Cl^−^ from the cytoplasm to the vacuole or their expulsion from the plant by inducing the expression of the ion transporter genes [30,31].

Salicylic acid has well-documented effects on photosynthetic activity, chlorophyll content, stomatal function, absorption of water, modulating mineral nutrition uptake, membrane stability, and transpiration rate [32,33]. It has also been found that it decreases the uptake of Na^+^ into roots and its subsequent transport to shoots while promoting K^+^ accumulation in shoots under salt stress [34]. It seems that SA plays a regulatory role in triggering biochemical enhancements in antioxidant defense in different plants, boosting the accumulation of osmotic regulators, including soluble sugars and proline, which subsequently results in lower levels of oxidative stress markers such as malondialdehyde (MDA) and hydrogen peroxide (H_2_O_2_), indicating reduced lipid peroxidation and better overall plant growth [35]. Thus, the accumulation of SA has been suggested as an endogenous marker for plant tolerance [36].

Jasmonic acid regulates several physiological processes and metabolite syntheses under environmental fluctuations [37,38]. JA is involved in stress resilience via enhancing the production of secondary metabolites [39]. There is growing evidence supporting the assumption that JA can significantly contribute to alleviating responses to abiotic stress [40]. For instance, there appears to be limited knowledge on how AMF and salinity influence endogenous ABA levels in plants. JA and SA have been shown to enhance a plant’s tolerance to salt stress; yet, their potential as candidates for promoting salinity tolerance in mycorrhizal plants remains underexplored.

*S. carnosa* Desf., a habitat-indifferent halophyte and an endemic extremophile species from Tunisia, *belonging to the Fabaceae family*, can thrive in both saline and salt-free environments [41]. *S. carnosa* grows in various climates across Tunisia, from semi-arid areas like “Kerker” to arid regions such as “Thelja” and “Douiret,” all characterized by alkaline–saline soils. It plays an essential role in forage and pastoral production, as it provides biomass for grazing while improving soil fertility. It is particularly effective in enriching the plant cover of soils affected by salinity [41]. In addition to these practical benefits, *S. carnosa* is an excellent model for studying plant–microorganism interactions in saline environments, deepening our understanding of how plants can thrive in challenging conditions.

Excessive accumulation of Na^+^ induces ionic and osmotic stress in *S. carnosa* plant cells [42]. A direct consequence of these primary effects is the increased production of H_2_O_2_, which induces lipid peroxidation of membrane lipids and, consequently, leads to a loss of membrane integrity [43]. This leads to reduced growth rates and productivity. However, AMF *Rhizophagus intraradices* inoculation was shown to effectively improve the salinity resistance of *S. carnosa* plants by enhancing their growth parameters and boosting the activities of enzymatic antioxidant defense systems [42,44]. *R. intraradices* is one of the most studied AMF species and easily propagates in pot culture [45]. This species not only improves plant growth through enhanced nutrient uptake but also contributes to soil fertility and boosts plants’ resistance to abiotic stress factors [46]. *R. intraradices* has the potential to influence defense mechanisms under salt stress conditions by modulating plant hormones. However, reports examining AMF-mediated regulation of hormones to enhance stress tolerance in *S. carnosa* plants are scarce. This study was conducted to examine whether inoculation with *R. intraradices* affects the hormone levels (ABA, SA, and JA) and nutrient acquisition capacity of *S. carnosa* plants under salt stress, providing novel insights into the role of AMF in mitigating the harmful effects on host plants through hormone regulation.

## 2. Materials and Methods

### 2.1. Plants and Fungal Inocula

The AM fungus species was *Rhizophagus intraradices* [47] EEZ 58 (Ri), which was obtained from the collection of “Estacion Experimental del Zaidin” (CSIC, Granada, Spain). *R. intraradices* was propagated with maize plants and preserved by storage in polyethylene bags at 4 °C. The fungal inoculum consisted of a mixture of soil, spores, hyphae, and mycorrhizal root fragments of maize.

Seeds of *S. carnosa* were collected from the locality of “Kalbia” (35°30′24.7″ N 10°08′29″ E) in Tunisia. These seeds were not subjected to any pre-treatment and were stored at 4 °C in the laboratory. Seeds were surface-sterilized by immersing them in a 0.5% sodium hypochlorite solution (*v*/*v*) for 5 min, followed by three rinses with sterile water. Half of the seeds were pre-germinated on vermiculite mixed with the inoculum, while the other half were placed on vermiculite without AMF inoculation, serving as non-inoculated controls. Both groups were maintained in a 28 °C environment for 10 days prior to being moved to plastic pots.

The growth substrate used was a 1:1 volume ratio of soil and quartz sand (<1 mm), which was sterilized by steaming at 100 °C for 1 h each day over three consecutive days to eliminate potential mycorrhizal spores and other microorganisms in the soil. Mediterranean loamy soil samples were gathered from the top layer (0–15 cm) in the province of Granada (Spain). The soil was air-dried, then sifted through a 2 mm sieve to eliminate root-stone residues, ensuring coherence. The properties of the sieved soil used for the growth substrate were as follows: pH 8.2 (measured in water, 1:5 *w*/*v*); 1.5% soil organic matter; 1 g kg^−1^ total phosphorus; 27 mg kg^−1^ available phosphorus (Olsen’s P); 1.9 g kg^−1^ total nitrogen; 0.69 g kg^−1^ available potassium; and 200 μS cm^−1^ electrical conductivity. Five hundred grams of this soil–sand mixture was placed into pots.

### 2.2. Experimental Design, Biological Treatments, and Growth Conditions

The experiment followed a factorial design with two factors (inoculation with AMF and salinity) and five replications, resulting in four treatments in total: (1) control plants (no treatment); (2) plants inoculated with *R. intraradices*; (3) salt stress plants in the absence of *R. intraradices*; and (4) salt stress plants in the presence of *R. intraradices*. Pre-germinated *S. carnosa* seeds were planted into a plastic pot containing 500 g of sterilized growth substrate. Five grams of AM fungal inoculum was added to each pot, two centimeters below the seeds, and the substrate was then covered. Control plants that were not inoculated received a filtrate of the inoculum to account for any variations in soil microbial communities, excluding AM fungi. The plants were grown in a greenhouse under conditions of 24/20 °C (day/night); 16 h/8 h light/dark photoperiod; 50–60% relative humidity; and an average photosynthetic photon flux density of 800 μmol m^−2^ s^−1^, as measured with a light meter (LICOR, model LI-188B, Lincoln, NE, USA). One month after transplanting *S. carnosa* seedlings to pots, plants were watered three times per week with 50 mL of either a 0 mM or 200 mM NaCl solution. To prevent osmotic shock, the saline solution was progressively raised by adding 50 mM NaCl each two days.

After 30 days of treatment, plants were harvested and divided into root and shoot parts, and their fresh weights were recorded. One portion was kept at −80 °C for biochemical analyses, while the rest was dehydrated in a forced-air oven at 70 °C for three days and weighed to assess biomass production.

### 2.3. Determination of Photosynthetic Pigment Concentrations

Photosynthetic pigments were extracted from five leaves per treatment using 0.1 g of fresh material in 5 mL of 80% aqueous acetone. The absorbance of the extracts was measured at 470, 646, and 663 nm to determine the concentrations of chlorophyll a (Chl. a), chlorophyll b (Chl. b), and total carotenoids (Car.), using a Hitachi U-1900 spectrophotometer (Hitachi Ltd., Tokyo, Japan). The pigment concentrations were calculated according to Lichtenthaler [48].

### 2.4. Hydrogen Peroxide Concentration

The concentration of leaf hydrogen peroxide (H_2_O_2_) was determined spectrophotometrically according to the method described by Aroca et al. [49]. Ca. 200 mg of fresh leaves from five plants per treatment was ground in liquid nitrogen and then homogenized with 5 mL of 5% (*w*/*v*) trichloroacetic acid (TCA) containing 0.1 g of activated charcoal and 1% (*w*/*v*) polivinilpolipirrolidona (PVPP). The homogenate was then centrifuged at 18,000× *g* for 10 min at 4 °C, and the supernatant was filtered through a Millipore filter (0.22 μm). An amount of 130 μL of supernatant was added to 1.2 mL of 100 mM potassium phosphate buffer (pH 8.4) and 0.6 mL of the colorimetric reagent. This reagent was prepared daily by mixing 1:1 (*v*/*v*) 0.6 mM 4-(2-pyridylazo) resorcinol (disodium salt) and 0.6 mM potassium titanium oxalate. H_2_O_2_ content of samples was measured by a spectrophotometer at 508 nm after being incubated for one hour at 45 °C.

### 2.5. Hormone Analysis

ABA accumulation in roots and shoots was quantified by a high-performance liquid chromatography electrospray mass spectrometry (HPLC–ESI–MS/MS) system following the protocol described by Aroca et al. [50].

Extraction, purification, and further analytical determination of SA were performed on the roots and shoots of three plants per treatment as follows: briefly, 0.2 g of frozen plant tissues (previously ground to a powder in a mortar with liquid N) was homogenized, in triplicate, with 50 μL of 1,000 ng ml−1 d4 salicylic acid in methanol and 2 mL of MeOH/H_2_O/HCOOH (90/9/1, *v*/*v*/*v*). After 1 h of mixing the samples at 2000 rpm using the Multi Reax shaker (Heidolph, Schwabach, Germany), the mixture was centrifuged at 15,000× *g* for 15 min at room temperature. A total of 0.5 mL of the supernatants was added to 0.3 mL of 0.2% acetic acid and centrifuged at 15,000× *g* for 10 min. The supernatant was later injected into an LC/MS system. SA was quantified by HPLC linked to a 3200 QTRAP LC/MS system (Applied Biosystems/MDS Sciex, Whitby, ON, Canada), equipped with a turbo ion spray interface. The detection and quantification of SA was performed by multiple reaction monitoring (MRM) in the negative-ion mode, employing multilevel calibration curves with deuterated d4 salicylic acid as an internal standard. The source parameters were curtain gas 20 psi; GS1 45 psi; GS2 50 psi; ion spray voltage of 4000 V, scan mode MRM; CAD gas medium; temperature 500 °C.

The extraction and purification of JA were carried out using the method described by Sánchez-Romera et al. [51]. A 0.2 g frozen sample of plant tissue from three plants per treatment as (previously ground to a powder in a mortar with liquid N) was homogenized, in triplicate, with 2 mL of methanol–water–formic acid (90:9:1, *v*/*v*/*v*). The deuterium-labeled internal standard (d2-MeJA) was added (50 μL of a stock solution of 1000 ng mL^−1^ of standard in methanol) to the extraction medium. The mixture was vortexed at 2000 rpm for 1 h using the Multi Reax shaker (Heidolph, Schwabach, Germany) and centrifuged at 12,000× *g* for 15 min at room temperature. For zero points, 0.5 mL of the supernatants was added to 0.3 mL of 0.2% acetic acid and centrifuged at 12,000× *g* for 15 min. Before the injection in the HPLC-ESIMS/MS system, the solution was centrifuged at 12,000× *g* for 10 min, and the supernatants were introduced into chromatographic vials. JA was quantified by HPLC-ESI-MS/MS using an HPLC device (2795 Alliance HT; Waters Co., Milford, MA, USA) linked to a 3200 Q TRAP LC/MS System (Applied Biosystems/MDS Sciex, ON, Canada), equipped with an electrospray interface. A reverse-phase column (Synergi 4 μm Hydro-RP 80A, 150 × 2 mm; Phenomenex, Torrance, CA, USA) was employed. A linear gradient of methanol (A) and 0.2% acetic acid in water (B) was employed: 60% A for 3 min, 60% A to 85% A in 9 min, 85% A for 1 min, and 85% A to 60% A in 1 min, followed by a stabilization time of 4 min. The flow rate was 0.20 mL min^−1^, the injection volume was 40 μL, and the column and sample temperatures were 20 °C. The detection and quantification of JA was carried out using multiple reaction monitoring (MRM) in the positive-ion mode, employing a multilevel calibration graph with deuterated hormone (d2-MeJA) as an internal standard.

The source parameters were curtain gas 20 psi; GS1 45 psi; GS2 50 psi; ion spray voltage-5000 V, scan mode MRM; CAD gas medium; temperature 500 °C.

### 2.6. Nutrient Content

Shoot concentrations of nitrogen (N) and carbon (C) were determined through mass spectrometry (ELEMENTAL LECO TruSpec CN) at the Analytical Service of the Instituto de Nutrición Animal, CSIC, located in Granada, Spain.

The concentrations of macronutrients (Ca, K, Mg, Na, and P) and micronutrients (Cu, Fe, Mn, and Zn) were determined in an extract digested with HNO_3_:HClO_4_ (2:1, *v*/*v*) using inductively coupled plasma optical emission spectrometry (ICP-OES; Varian I.C.P. 720-ES) conducted by the Instrumentation Service at the Estación Experimental del Zaidín (CSIC), Granada, Spain.

### 2.7. Statistical Analysis

Data were analyzed using the SPSS 20 software package for Windows and were subjected to two-way general linear model ANOVA (analysis of variance) to assess the effects of each treatment. Post hoc comparisons of means were performed using Duncan’s [52] multiple range test at *p* < 0.05. Differences were considered significant at *p* < 0.05. Principal component analysis (PCA) was performed using XLSTAT software version 2014, with variables centered around their means and standardized to a standard deviation of 1.

## 3. Results

### 3.1. Biomass Yield

**Figure 1:**
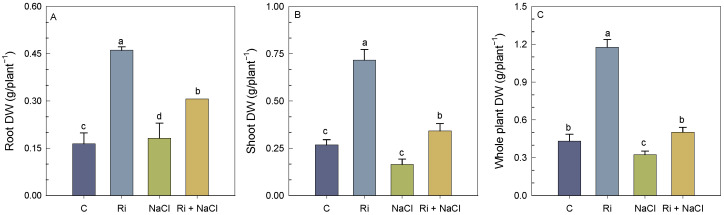
Effect of *R. intraradices* inoculation on shoot dry weight (DW) (**A**), root dry weight (DW) (**B**), and whole plant dry weight (DW) (**C**) of *S. carnosa* plants, challenged or not, for 4 weeks with 200 mM NaCl. Values are means of five replicates ±/SE and different letters indicate a significant difference (*p* < 0.05) using Duncan’s test. C: control treatment; Ri: *R. intraradices* treatment; NaCl: NaCl treatment; Ri + NaCl: *R. intraradices* + NaCl treatment.

As shown in Figure 1A–C, salt treatment significantly affected the shoot dry weight (DW) and total DW parameters by 38% and 20%, respectively, in the non-mycorrhizal plants; however, the inoculated plants grew better than the non-inoculated plants under the control as well as saline conditions. AMF symbiosis mitigated the detrimental effect of salinity by increasing the shoot DW, root DW, and total DW of *S. carnosa* by 107%, 67%, and 87%, respectively, compared to the NaCl-stressed plants. These results indicated that mycorrhizal symbiosis greatly improved the survival of *S. carnosa* plants under salt stress conditions.

### 3.2. Photosynthetic Pigment Concentrations

**Figure 2:**
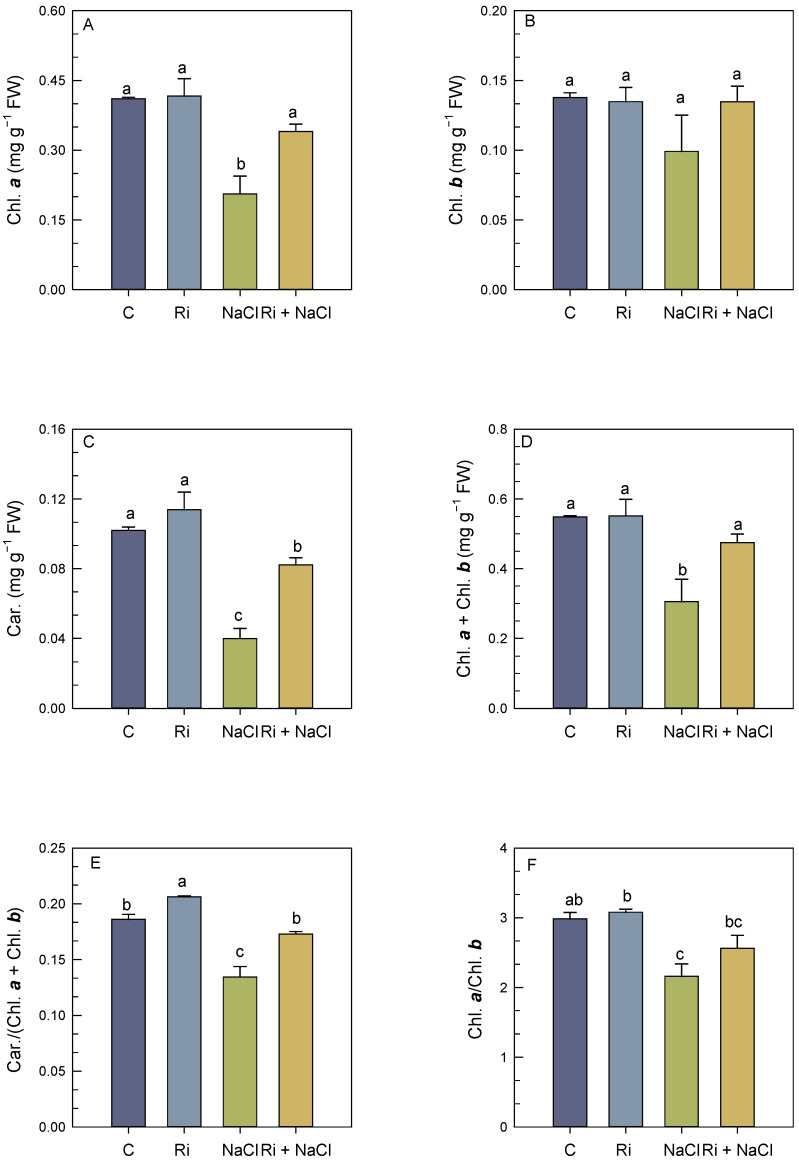
Effect of *R. intraradices* inoculation on photosynthetic pigment contents in shoots of *S. carnosa* plants, challenged or not, for 4 weeks with 200 mM NaCl. Chl. *a*: chlorophyll a (**A**); Chl. *b*: chlorophyll b (**B**); Car.: carotenoid (**C**); Chl. *a* + Chl. *b*: chlorophyll a + chlorophyll b (**D**); Chl. *a*/Chl. *b*: chlorophyll a/chlorophyll b (**E**); Car./(Chl. *a* + Chl. *b*): carotenoid/(chlorophyll a + chlorophyll b) (**F**). Values are means of five replicates ±/SE and different letters indicate a significant difference (*p* < 0.05) using Duncan’s test. C: control treatment; Ri: *R. intraradices* treatment; NaCl: NaCl treatment; Ri+NaCl: *R. intraradices* + NaCl treatment.

Salt stress treatment resulted in a significant decrease in the concentrations of Chl. *a*, Chl. *a* + Chl. *b*, and carotenoids (Car.), whereas in the AMF mycorrhizal plants, the values of these parameters were restored (Figure 2A–D). AMF treatment enhanced Chl. *a*, Chl. *b*, Chl. *a* + Chl. *b*, and Car. by 65%, 36%, 56%, and 105%, respectively, as compared to the salt-affected plants (Figure 2A–D).

Plants exposed to salinity showed less Car./(Chl. *a* + Chl. *b*) and Chl. *a*/Chl. *b* ratios as compared to the control plants, while mycorrhizal symbiosis alleviated the negative effect by improving those parameters and showed a ratio similar to that of the control plants (Figure 2E,F).

### 3.3. Contents of Nutrients

Compared with the control, salt treatment highly increased the shoot Na^+^ concentration in both the non-mycorrhizal and mycorrhizal plants, while the AMF plants exhibited a lower Na^+^ content than the non-AMF plants. Specifically, AMF demonstrated its protective effects by reducing the sodium content in the shoots of the salt-affected plants by 23%, although the concentration of this ion was unaffected by mycorrhizal symbiosis under salt-free conditions (Table 1).

Salinity treatment significantly reduced the shoot C, N, K, Mg, S, Zn, and Cu concentrations by 9.2%, 22.2%, 46.4%, 18.2%, 49.7%, 31%, and 25%, respectively, compared to the control plants. In contrast, mycorrhizal symbiosis increased the concentrations of C, N, K, P, S, Cu, and Zn by 14.5%, 22.6%, 13.5%, 41.8%, 17%, 26.4%, and 26%, respectively, compared to the non-mycorrhizal plants. However, it did not significantly affect the content of these elements under salt-free conditions (Table 1).

### 3.4. H_2_O_2_ Content

**Figure 3:**
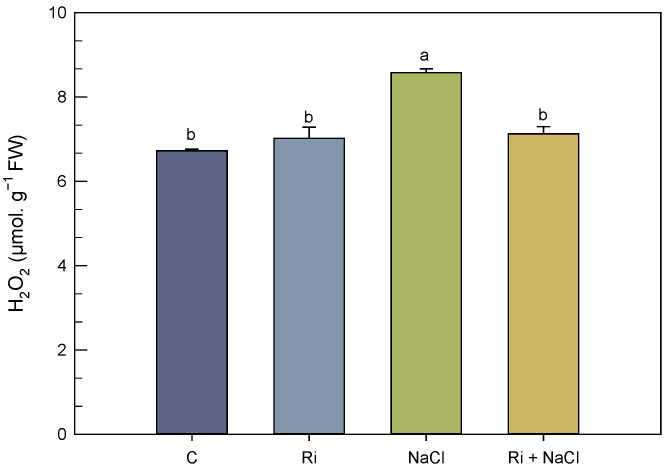
Effect of *R. intraradices* inoculation on shoot hydrogen peroxide content of *S. carnosa*, challenged or not, for 4 weeks with 200 mM NaCl. Different letters indicate significant differences between treatments (*p* < 0.05). Values are means of five replicates ±/SE and different letters indicate a significant difference (*p* < 0.05) using Duncan’s test. C: control treatment; Ri: *R. intraradices* treatment; NaCl: NaCl treatment; Ri + NaCl: *R. intraradices* + NaCl treatment.

Hydrogen peroxide generation was measured in *S. carnosa* plants to evaluate salt-induced oxidative stress (Figure 3). In the non-AMF plants, the shoot H_2_O_2_ content increased by 27.67% as compared to the non-saline treatment under salt stress conditions. However, mycorrhizal symbiosis significantly reduced this level of H_2_O_2_ by 17% (Figure 3). Under salt-free conditions, the H_2_O_2_ content showed no significant difference between the mycorrhizal and non-mycorrhizal *S. carnosa* plants.

### 3.5. Endogenous Phytohormone Status

**Figure 4:**
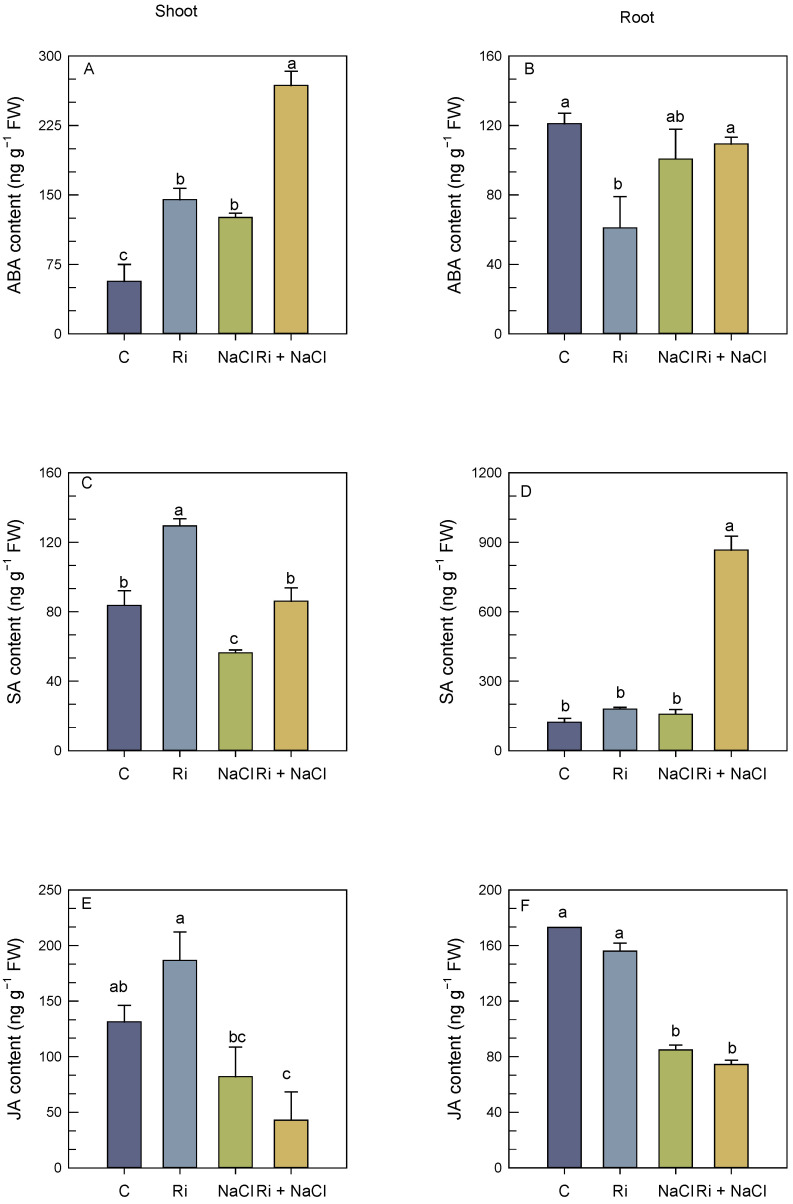
Effect of *R. intraradices* inoculation on abscisic acid (ABA), salicylic acid (SA), and jasmonic acid (JA) contents in shoots (**A**,**C**,**E**) and roots (**B**,**D**,**F**) of *S. carnosa* plants, challenged or not, for 4 weeks with 200 mM NaCl. Values are means of three replicates ±/SE and different letters indicate a significant difference (*p* < 0.05) using Duncan’s test. C: control treatment; Ri: *R. intraradices* treatment; NaCl: NaCl treatment; Ri + NaCl: *R. intraradices* + NaCl treatment.

The endogenous levels of plant stress hormones, including abscisic acid (ABA), jasmonic acid (JA), and salicylic acid (SA), under various treatments are presented in Figure 4.

Under salt-free conditions, AMF increased the ABA level by 155% in the shoots but reduced its accumulation in the roots. As shown in Figure 4A, salt stress significantly increased the ABA levels in the shoots by 121% relative to the controls. However, salt stress did not alter the ABA levels in the roots as compared to the control plants. AMF sharply increased the ABA levels by 113% in the shoots, relative to those seen for salt stress without inoculation, whereas it did not significantly change the ABA levels in the roots relative to the stressed plants (Figure 4A,B).

AMF and salt stress significantly affected the endogenous SA levels, particularly in the shoots (Figure 4C). In the absence of salt stress, AMF inoculation increased the SA levels in the shoots by 55% as compared to the control plants but had no significant effects on the SA levels of the roots (Figure 4A,D). However, a decrease in the shoot SA level by 32% was observed in the salt-affected plants, with respect to the control plants (Figure 4C), whereas no significant variation in the root SA content was observed between the control and salt-affected plants (Figure 4D). Under salt stress conditions, AMF strongly increased the SA levels in the roots (4.5-fold) relative to the stressed plants (Figure 4D). Additionally, AMF inoculation mitigated salt-induced decreases in the SA levels in the *S. carnosa* shoots grown under salt stress conditions: the SA content in the inoculated AMF plants was 52% higher than in the stressed non-mycorrhizal plants (Figure 4C).

The JA content decreased by nearly 51% in the roots of the *S. carnosa* salt-affected plants as compared to the control plants (Figure 4F). In the absence of salt, no significant variation in the JA shoot content was observed between the non-mycorrhizal and mycorrhizal plants, whereas a significant decrease was observed in the roots of the AMF mycorrhizal plants (Figure 4C,D). Under salt stress conditions, AMF mycorrhizal did not change significantly the JA content of both the shoots as well as the roots (Figure 4C,D). There were no significant differences in the JA content between the non-inoculated and inoculated shoots under salt stress conditions (Figure 4C).

### 3.6. Principal Component Analysis

All studied parameters and the different treatments are projected onto the F1–F2 principal factorial plane that explains 71.89% of the variation for the control and R. intraradices and 82.14% of the variation for the NaCl and NaCl + R. intraradices treatments.

**Figure 5:**
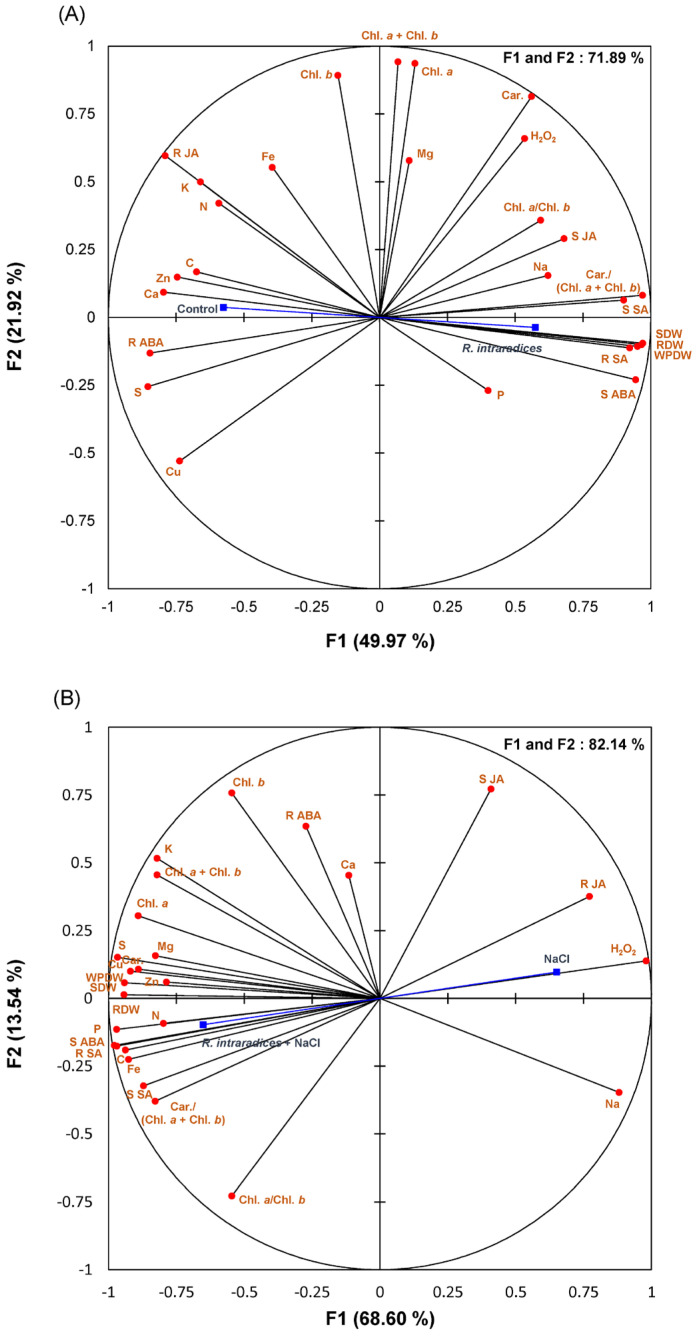
Principal component analysis (PCA). Red circles (⬤) represent various analysis parameters, while blue squares (◼) denote different treatments: control and *R. intraradices*—(**A**); NaCl and NaCl + *R. intraradices*—(**B**). S DW—root dry weight; R DW—root dry weight; WP DW—whole plant dry weight; Ch. *a*—chlorophyll *a*; Chl. *b*—chlorophyll *b*; Car—carotenoid; S ABA—shoot abscisic acid content; R ABA—root abscisic acid content; S SA—shoot salicylic acid content; R SA—root salicylic acid content; S JA—shoot jasmonic acid content; R JA—root jasmonic acid content.

Under salt-free conditions, several significant positive correlations were observed with AMF symbiosis, including the shoot dry weight (SDW), root dry weight (RDW), whole plant dry weight (WPDW), shoot abscisic acid (S ABA) content, and shoot salicylic acid (S SA) content. These correlations suggest that microbial treatment generally enhances these parameters (Figure 5A).

In contrast, the NaCl treatment showed positive correlations with the Na+ and H_2_O_2_ content, indicating an increase in these parameters compared to the control, followed by a decrease in their levels with AMF symbiosis, as reflected in the negative correlations (Figure 5B).

Under salt stress conditions, numerous significant positive correlations were found between AMF symbiosis and various parameters, including the shoot dry weight, root dry weight, whole plant dry weight, chlorophyll a, total chlorophyll, carotenoid contents, shoot abscisic acid content, and shoot and root salicylic acid contents, as well as the concentrations of C, N, K, P, S, Cu, and Zn in the shoot (Figure 5B).

## 4. Discussion

Owing to their sessile nature, plants face various challenges, such as biotic and abiotic stresses (such as salinity) [53]. Arbuscular mycorrhizal fungi (AMF) can improve plant fitness by bolstering plant resistance/tolerance against salt stress conditions [54]. The present research was performed to further improve our understanding of the physiological mechanisms involved in the induction of such processes through AMF colonization for lowering salt-induced reductions in the endemic Tunisian halophyte *S. carnosa* growth. Several studies have shown that salinity can inhibit plant growth and development by disrupting various physiological and biochemical mechanisms [55]. Plant biomass accumulation and allocation reflect a plant resource acquisition strategy and ecological adaptation, with patterns influenced by environmental changes [56].

Our findings indicated that exposing plants to salt stress led to significant reductions in the shoot DW and total DW. The primary factors behind the reduced growth of plants under stress are the decreased rates of both cell division and cell elongation [57]. In our study, we observed that *R. intraradices* mycorrhizal plants showed better growth prospects in comparison with non-mycorrhizal plants under both salt-free and salinized conditions. *R. intraradices* effectively mitigated the adverse effect of salinization on the growth of this facultative halophyte. AM fungi have been found to boost plant growth in different halophyte species, such as in *Suaeda glauca*, *Kosteletzkya virginica*, *Aeluropus littoralis*, *Puccinellia distans*, *Suaeda salsa*, and *Asteriscus maritimus*, especially under high salt concentrations [58,59,60,61,62,63]. Mycorrhizal symbiosis may lead to the growth of and improvement in host plants because mycorrhizal extraradial hyphae acquire more water and nutrients [64].

The results from the present study showed that salinity leads to an increase in Na⁺ content in plants, as they tend to absorb more Na^+^ when soil salt concentrations are higher. Salt toxicity occurs when an accumulation of Na^+^ accumulates to excessive levels inside plant cells, particularly in the leaves, where the majority of metabolic processes occur, ultimately affecting the plant’s growth and development [65]. Our findings also showed that the shoot N, K, Cu, and Zn contents were reduced by salt stress, which may be due to the antagonism interaction between toxic ions and essential nutrients and further leads to their immobilization. Salt stress caused ion imbalance in *S. carnosa* plants, which could also be a reason behind reduced growth.

Nitrogen (N) is the most important element to influence plant growth, development, and overall productivity out of all the required nutritious compounds because N is part of amino acids, which are the building blocks of proteins and enzymes that are involved in plant salt tolerance through different mechanisms [66]. Salinity has been reported to affect nitrogen metabolism primarily by reducing N uptake, alteration in the activities of N assimilating enzymes, decreasing amino acid production, and enhancing the activity of hydrolyzing enzymes [67].

AMF have been found to enhance mineral element acquisition by glycophytes and halophytes, a mechanism widely considered crucial for boosting plant salt tolerance [14]. The findings of this research demonstrate that *R. intraradices* significantly increased nutrient uptake, particularly the uptake of N and P in the shoots of *S. carnosa* grown under salt stress conditions. AMF enhance nutrient acquisition by forming an extensive hyphal network, effectively increasing the root’s access to a larger soil volume [6]. Furthermore, Gupta et al. [68] also suggested that fungi enhance gene expression and upregulate the activity of various ion transporters, leading to better nutrient uptake. Enhanced nitrate uptake in mycorrhizal plants is attributed to the ability of AMF to preserve membrane stability and boost nitrate reductase activity [69]. The mobility of phosphorus in soils is low, but AMF may enhance plant P uptake through several mechanisms: expanding the P depletion zone, maintaining phosphate concentration by forming polyphosphates, and expressing high-affinity phosphate transporters. AMF can accumulate more P than roots, sustaining its movement into plants. According to Liu et al. [70], higher shoot P causes an increase in the sink size of Cu and Zn, which in turn triggers nutrient uptake and translocation to shoots. In addition, mycorrhizal colonization in *S. carnosa* plants has been found to limit the uptake of Na^+^ while improving K^+^ absorption under saline conditions. Mycorrhizal plants have the ability to manage the translocation of Na^+^ to aboveground parts and regulate their internal concentrations [71]. This is due to their ability to exclude Na^+^ out of the cytosol or sequester it in vacuoles. As a result, AM plants sustain a higher K^+^:Na^+^ ratio, which helps prevent the disruption of cellular enzymatic activities and the inhibition of protein synthesis [13]. Furthermore, the AM symbiosis interaction is capable of absorbing vital nutrients like essential minerals while preventing the uptake of Na^+^. Thus, mycorrhizae can help mitigate nutrient deficiencies, thus playing a vital role in enhancing the ability of host plants to withstand the negative impacts of salinization.

The results triggered from the current study demonstrate that salt stress results in a decrease in the levels of photosynthetic pigments (Chl. *a*, Chl. *b*, and carotenoids) in *S. carnosa* plants. Reduced photosynthetic pigment levels in *S. carnosa* under salt stress may be attributed to decreased uptake of essential minerals like magnesium and nitrogen, which are vital for chlorophyll biosynthesis [72]. Interestingly, inoculation with *R. intraradices* significantly elevates the photosynthetic pigment contents in *S. carnosa* plants under salt treatment. In the current investigation, increased N concentrations in the shoots of *R. intraradices*-inoculated plants were accompanied by an increase in chlorophyll pigment levels.

Phytohormones play crucial roles in regulating various physiological and biochemical processes that manage plant growth and development under both normal and stressed conditions and thus also referred to as growth regulators [73]. Recent studies highlighted the role of phytohormones in imparting salt stress tolerance in plants [74,75]. Although there is extensive research on the function of phytohormones in salinity tolerance, there has been little focus on this aspect when investigating AMF-induced salinity tolerance in plants. Additionally, it is well known that during AM symbiosis, ABA, JA, and SA serve as signaling molecules [76,77]. Consequently, it is considered that these hormones are crucial in boosting plants’ ability to tolerate salt stress.

ABA converts environmental signals into gene expression changes to adapt to abiotic stress conditions [78]. Mycorrhizal *S. carnosa* had a higher shoot ABA content compared with non-mycorrhizal plants under salt stress conditions, which could be associated with priming to improve salinity tolerance. Salt stress had no effect on the content of ABA in *S. carnosa* roots, which is consistent with a salt-resistant maize cultivar [79]. Therefore, the higher endogenous abscisic acid content in *S. carnosa* shoots under salt stress possibly represents higher salt tolerance of mycorrhizal *S. carnosa* plants. Ren et al. [80] have shown that the higher level of endogenous ABA in AMF plants protects them from salinity by inducing the synthesis of strigolactones via H_2_O_2_ signaling.

Additionally, it has been demonstrated that AMF colonization improves the endogenous concentrations of SA and JA [81]. However, studies on the relationship between the effect of AMF on the induction of those stress hormones and the improvement in salt stress tolerance are scarce.

SA, a phenolic endogenous growth regulator, serves as a crucial signaling molecule in plants and influences physiological processes like growth, photosynthesis, and nitrogen metabolism to have a variety of effects on tolerance to biotic and abiotic stress [82,83]. The present finding showed that *R. intraradices* boosted the concentrations of SA in both the shoot and root in *S. carnosa* under salt stress conditions as compared to stressed plants. Nazar et al. [82] have shown that SA takes part in the removal of excess ROS and protects plants from oxidative damage. SA’s role in protecting against oxidative stress is played by its ability to induce antioxidant enzyme expression [84,85]. SA acts by increasing the pigment content and photosynthetic rate, preserving membrane stability, and decreasing Na^+^ accumulation in plants [86]. The beneficial effects of SA on photosynthetic capacity can be attributed to its stimulating effects on RuBisCO activity and pigment contents [87]. Mycorrhizal *S. carnosa* plants showed higher photosynthetic pigment concentrations compared to the stressed plants.

Previous studies have examined the effects of nitrogen on photosynthetic enzymes and other elements of photosynthetic metabolism [88,89]. The protective function of SA in maintaining membrane integrity and regulating ion uptake has also been previously reported [90]. Consequently, mycorrhizal plants may be effectively protected from excessive salinity by having high amounts of SA accumulated under salt stress conditions.

Jasmonic acid (JA) is an endogenous growth-regulating substance, initially described as part of a defense mechanism when plants are exposed to biotic and abiotic stresses [38]. According to Pedranzani et al. [91], the highest concentrations of JA in salinity-tolerant tomato plants could act as a protective mechanism against salinity. In contrast, the JA levels remained unaltered in the shoots of mycorrhizal *S. carnosa* plants compared with non-inoculated plants. This can be attributed to the fact that JA exerts its effects at a very early stage. It seems possible that AMF may exert its protective effect partially through the increased levels of SA and ABA. According to Gupta et al. [92], it appears that SA and JA interact antagonistically, and the SA/JA ratio has been proposed as a potential indicator of saline stress tolerance [17]. Under salt treatment, AMF may change the endogenous hormone balance, such as ABA and SA, which provides a significant hint for understanding the protection mechanisms against salt stress.

## 5. Conclusions

The results of this investigation showed that AM symbiosis significantly increased the biomass production of *S. carnosa* grown in salt stress conditions. AM fungal symbiosis contributes to the protection of plants against salt stress through increasing host plant nutrition (C, N, K, P, Cu, and Zn) and modulating the accumulation of the endogenous hormones ABA, SA, and JA in both roots and shoots. SA and ABA contribute to the mitigation of salinity-induced damage and are associated with improved salt resistance and/or tolerance in this species. In *S. carnosa* plants that were exposed to salt stress, SA and ABA seemed to interact synergistically and have antagonistic effects on the JA levels. According to this study, the successful symbiotic relationship between *R. intraradices* and *S. carnosa* can promote the growth of *S. carnosa* in saline agricultural soils. These findings open up new avenues for promoting growth in saline soils, with potential applications of arbuscular mycorrhizal fungi (AMF) in field trials and restoration projects aimed at rehabilitating degraded areas. Finally, investigating the molecular mechanisms underlying the observed effects is essential to validate these findings and confirm the biochemical pathways involved.

## Figures and Tables

**Table 1:** Effect of *R. intraradices* inoculation on nutrient shoots content of *S. carnosa* plants, challenged or not, for 4 weeks with 200 mM NaCl. Values are means of five replicates ±/SE and different letters indicate a significant difference (*p* < 0.05) using Duncan’s test.

	0 mM NaCl		200 mM NaCl	
	Control	*R. intraradices*	Stressed	*R. intraradices*
Macroelements (mmol/g)				
C	28.3 ± 0.57 ab	27.0 ± 0.45 bc	25.7 ± 0.16 c	29.4 ± 0.7 a
N	2.39 ± 0.10 a	2.11 ± 0.08 ab	1.86 ± 0.04 b	2.28 ± 0.14 a
Ca	0.6 ± 0.03 a	0.6 ± 0.08 a	0.51 ± 0.01 a	0.53 ± 0.05 a
K	1.28 ± 0.03 a	1.19 ± 0.02 b	0.68 ± 0.03 d	0.77 ±0.02 c
Mg	0.49 ± 0.032 a	0.49 ± 0.026 a	0.40 ± 0.002 b	0.43 ±0.009 ab
Na	0.10 ± 0.01 c	0.22 ± 0.08 c	2.73 ± 0.11 b	2.41 ± 0.01 a
P	0.048 ± 0.001bc	0.053 ± 0.005 ab	0.043 ± 0.001 c	0.061 ± 0.001 a
Microelements (µmol/g)				
S	0.80 ± 0.043 a	0.68 ± 0.032 b	0.40 ± 0.019 c	0.47 ± 0.014 c
Cu	0.14 ± 0.007 a	0.13 ± 0.011 a	0.10 ± 0.005 b	0.12 ± 0.004 a
Fe	3.72 ± 0.66 bc	3.02 ± 0.28 c	4.7 ± 0 ab	5.57 ± 0.19 a
Zn	0.89 ± 0.02 a	0.64 ± 0.07 b	0.67 ± 0.03 b	0.84 ± 0.04 a

## Data Availability

All relevant data can be found in the manuscript.
